# The Pharmacokinetics and Pharmacodynamics of Lidocaine-Loaded Biodegradable Poly(lactic-*co*-glycolic acid) Microspheres

**DOI:** 10.3390/ijms151017469

**Published:** 2014-09-29

**Authors:** Jianming Liu, Xin Lv

**Affiliations:** Department of Anesthesiology, Shanghai Pulmonary Hospital, Tongji University School of Medicine, 507 Zhengmin Road, Yangpu District, Shanghai 200433, China; E-Mail: liujianming@126.com

**Keywords:** lidocaine, PLGA, microspheres, pharmacokinetics, pharmacodynamics

## Abstract

The purpose of this study was to develop novel lidocaine microspheres. Microspheres were prepared by the oil-in-water (o/w) emulsion technique using poly(d,l-lactide-*co*-glycolide acid) (PLGA) for the controlled delivery of lidocaine. The average diameter of lidocaine PLGA microspheres was 2.34 ± 0.3 μm. The poly disperse index was 0.21 ± 0.03, and the zeta potential was +0.34 ± 0.02 mV. The encapsulation efficiency and drug loading of the prepared microspheres were 90.5% ± 4.3% and 11.2% ± 1.4%. *In vitro* release indicated that the lidocaine microspheres had a well-sustained release efficacy, and *in vivo* studies showed that the area under the curve of lidocaine in microspheres was 2.02–2.06-fold that of lidocaine injection (*p* < 0.05). The pharmacodynamics results showed that lidocaine microspheres showed a significant release effect in rats, that the process to achieve efficacy was calm and lasting and that the analgesic effect had a significant dose-dependency.

## 1. Introduction

Lidocaine is an amide-type of local anesthetic. It is the preferred drug to prevent acute myocardial infarction, various heart diseases complicated by rapid ventricular arrhythmias, premature ventricular contractions of acute myocardial infarction, ventricular tachycardia and room tremor [1]. Oral administration of lidocaine has low bioavailability and a relatively strong liver first pass effect. After intramuscular injection, it is completely absorbed and could be quickly absorbed in the heart, brain, kidney and other tissues with a rich blood supply. The apparent volume of distribution was approximately 1 L/kg; the protein binding rate was about 51%. It is immediately effective after intravenous injection (about 45 to 90 s), for 10 to 20 min, *T*_1/2α_ (distribution half-life) of 10 min, *T*_1/2β_ (elimination half-life) about 1 to 2 h [2]. Clinically, in order to maintain an effective therapeutic concentration, frequent small doses of lidocaine injections to patients are required, which cause both pain and inconvenience to patients and lead to side effects, because of the accumulated blood concentration. In recent years, scholars have conducted a series of studies on local anesthetic sustained release delivery systems. These made sustained and controlled release formulations to extend the single-dose duration of the analgesic effect, to reduce the frequency of administration and to improve application compliance, while reducing fluctuations in the plasma concentration and drug toxicity, such as liposomes, implants, microspheres, *etc*. [3,4,5,6,7,8].

Poly(d,l-lactide-*co*-glycolide acid) (PLGA) copolymers have been developed in the past 10 years. These are a high polymer polymerized by polylactic acid and glycolic acid monomers with different proportions. It is non-toxic, non-irritating and fully biodegradable with good biocompatibility and human adaptability. *In vivo*, the final degradation product of PLGA is lactate, which can be metabolized by intravital cells. It can be eventually completely degraded into carbon dioxide and water and be exhausted out of the body. It is safe and will not cause a significant inflammatory response, immune response and cell toxicity. It has the advantage of self-degradation *in vivo* and being excreted, to avoid secondary damage to the patient. It is a biodegradable carrier material with good biocompatibility [9].

In order to prolong the effective time of lidocaine, to reduce the frequency of administration and to reduce side effects, this article uses PLGA microsphere technology to prepare lidocaine release microspheres. *In vivo* evaluations of characterization, release, pharmacokinetics and pharmacodynamics were conducted in order to provide the pharmacokinetic parameters for the further study of lidocaine sustained-release preparations.

## 2. Results and Discussion

**Figure 1:**
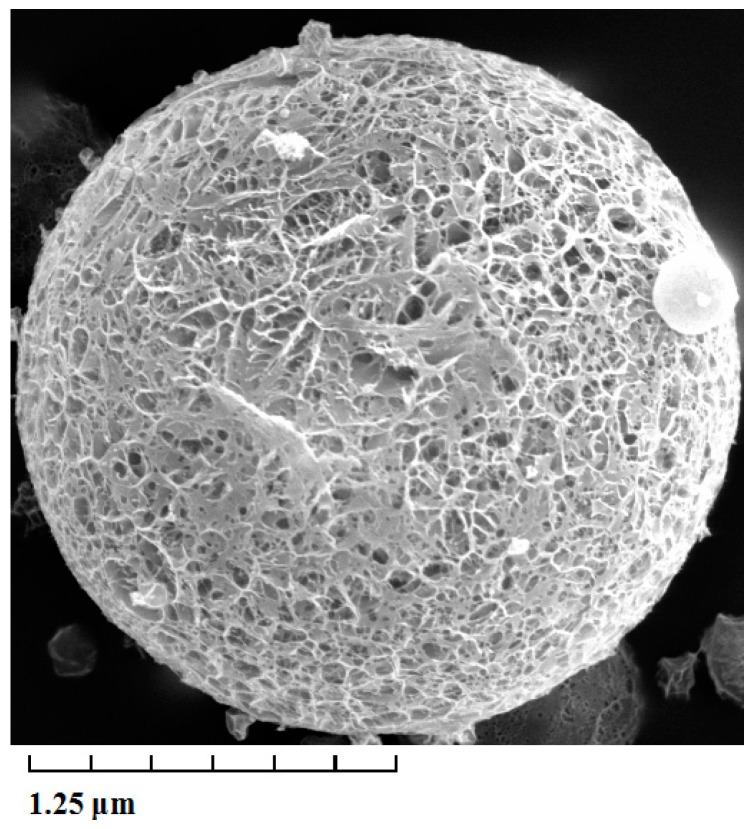
Transmission electron microscope photograph of lidocaine-loaded poly(d,l-lactide-*co*-glycolide acid) (PLGA) microspheres. Magnification × 5000.

The average diameter, as well as the size distribution of lidocaine-loaded PLGA microspheres were calculated by direct measurement in a NICOMP 380 Submicron Particle Sizer (Santa Barbara, CA, USA). The average diameter of lidocaine PLGA microspheres was 2.34 ± 0.3 μm. The poly disperse index (PDI) was 0.21 ± 0.03 μm, and the zeta potential was +0.34 ± 0.02 mV. As shown in Figure 1, the surface morphology of lidocaine microspheres was observed by transmission electron microscope (TEM). The microspheres were spherical in shape with a smooth surface, and the size was uniform and appropriate for administration via intravenous injection. The encapsulation efficiency and drug loading of prepared microspheres were 90.5% ± 4.3% and 11.2% ± 1.4% (Sample number = 3), respectively. The stability of lidocaine microspheres in phosphate buffer at 37 °C was studied before the drug release experiments were carried out. The stability data of lidocaine microspheres showed that when stored at 37 °C for 48 h, the surface morphology and content of lidocaine had no notable changes.

**Figure 2:**
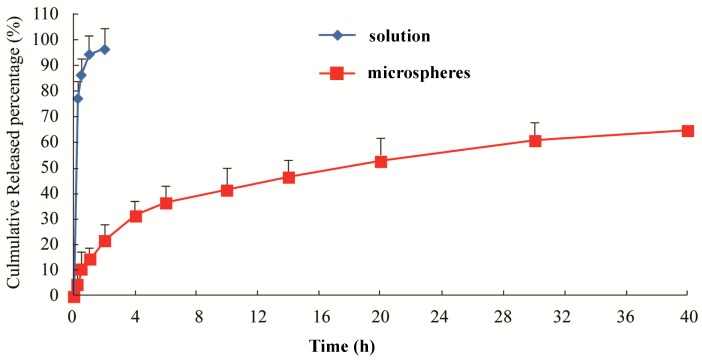
*In vitro* release of the lidocaine microspheres in human serum albumin (HSA).

Figure 2 shows that, compared with the raw material drug, the release of lidocaine microspheres had a significant slow-release effect in the plasma. The raw material drug released completely at around 1 h (93%), while the release of microspheres only reached 51% within 20 h. In the following 4 h, the microspheres entered the slow release period and released up to about 61% at the end of the observation (40 h). During the release process, the release of microspheres showed two distinct phases: rapid release during the first 4 h, with basically no sudden release phenomenon; and then, this was stabilized. The cumulative release of drug from the microspheres was fit by a single exponential function, Weibull function and Higuchi model. The correlation coefficient for each equation was used as the index. It was found that *in vitro* release of lidocaine microspheres was more in line with the Higuchi model, which proved that microsphere with PLGA as a carrier had better sustained release. The large ratio of surface area and volume of lidocaine, as well as surfactant promoted rapid drug release, with a later slow continuous drug release with the gradual dissolution of the PLGA skeleton of microspheres. Part of the drug was adsorbed onto the shallow surface or existed in free drug form, with most of the drug wrapped in the PLGA skeleton.

**Figure 3:**
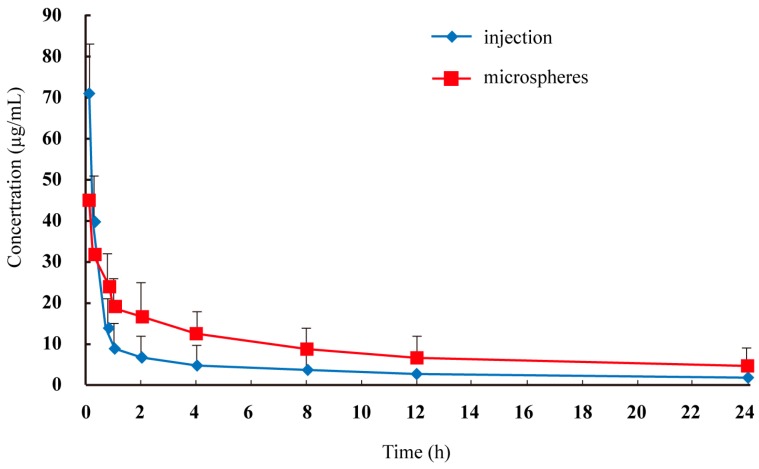
Mean plasma lidocaine concentration in rats after intravenous administration of two formulations (*n* = 6).

The plasma concentration-time profiles of lidocaine after intravenous administration by injection and microspheres to rats are shown in Figure 3, and the pharmacokinetic parameters are summarized in Table 1. The area under the curve of lidocaine in microspheres was 2.02–2.06-fold that of lidocaine injection (*p* < 0.05). The maximum plasma concentration of lidocaine injection was 1.58-fold that of lidocaine microspheres (*p* < 0.05). The relatively slower time to maximum plasma concentration of lidocaine microspheres suggested a sustained-release profile *in vivo*, which was consistent with the results of the *in vitro* release study.

**Table 1:** Pharmacokinetic parameters of lidocaine in rats after intravenous administration of two formulations (*n* = 6).

Group	*T*_1/2_ (h)	*C*_max_（μg·mL^−1^）	*T*_max_ (h)	*AUC*_0-T_ (h·µg·mL^−1^)	*AUC*_0-∞_ (h·µg·mL^−1^)
Injection	1.2 ± 0.3	72.4 ± 12.3	0.083	110.6 ± 32.1	129.7 ± 35.8
Microspheres	2.6 ± 0.7 *	45.7 ± 8.4 *	0.083	223.7 ± 45.2 *	267.3 ± 52.5 *

* *p* < 0.05: lidocaine PLGA microspheres *vs*. lidocaine injection.

Table 2 showed the pharmacodynamics results of lidocaine. From the results, the lidocaine solution group could quickly achieve an analgesic effect: the writhing inhibition rate after 1 h of administration reached 94.4%. However, the action period was limited, and the value reduced to 23.8% when observed in 2 h. On the contrary, the lidocaine microsphere groups of high, medium and low doses had a significant analgesic effect compared with the control group. Around 4 h of administration, they had the most significant effect: the maximum writhing inhibition rate of three groups (high, medium and low doses) were, respectively, 57.8%, 50.6% and 38.8%. Meanwhile, the analgesic effect duration of three groups was 6–8 h, which was longer compared with the solution group (4 h). Lidocaine microspheres showed a significant release effect in rats; the process to achieve efficacy was calm and lasting and the analgesic effect had a significant dose-dependency.

**Table 2:** The analgesic efficacy of lidocaine microspheres and lidocaine injection on rats.

Time (h)	Response Inhibition (RI) %			
	Lidocaine Injection (4 mg/kg)	High Dose Microspheres (10 mg/kg)	Middle Dose Microspheres (4 mg/kg)	Low Dose Microspheres (2.5 mg/kg)
0.5	69.5 ± 3.9	30.2 ± 3.5 *	24.3 ± 2.9 *	17.2 ± 2.6 *
1	94.4 ± 5.2	41.2 ± 5.4 *	35.2 ± 3.7 *	24.3 ± 2.8 *
2	23.8 ± 7.5	53.6 ± 6.3 *	44.2 ± 5.1 *	32.7 ± 3.3
4	10.5 ± 2.4	57.8 ± 9.2 *	50.6 ± 5.3 *	38.8 ± 4.1 *
6	6.4 ± 1.7	46.2 ± 3.9 *	37.4 ± 2.7 *	29.4 ± 2.4
8	2.1 ± 1.2	22.8 ± 3.7 *	16.1 ± 2.5 *	11.4 ± 1.6
10	1.9 ± 0.6	11.7 ± 1.5 *	7.3 ± 1.1 *	5.4 ± 0.7

* *p* < 0.05: lidocaine PLGA microspheres *vs*. lidocaine injection.

The amount of lidocaine-loaded PLGA microspheres was 90.5 mg of the drug per ten milligrams of microspheres. This percentage of entrapment efficiency was very high due to the chemical-physical characteristics of the drug and the preparation method used. The high solubility of both lidocaine and PLGA in dichloromethane allowed for obtaining a solution that can be sprayed through the nozzle of a spray-dryer. The use of polymer and drug solutions improved the entrapment efficiency of drugs with regard to the results obtained when the drug was not soluble in the same solvent as the polymer. Thus, the entrapment efficiency of BSA in poly(l-caprolactone) microspheres was about 43% in the absence of an emulsion stabilizer [10]. On the contrary, the entrapment efficiency of ketoprofen-loaded poly(l-caprolactone) microspheres was about 97%, since both compounds were soluble in the organic solvent [11].

This study used a protein precipitation method to process plasma samples and required less samples. Compared with organic solvent extraction in literature reports [12,13], it was simpler and more suitable for the mass analysis of biological samples; and it satisfied the sensitivity of this study. Thus, the protein precipitation method was used as the pretreatment method for lidocaine *in vivo* sample determination. Meanwhile, through comparison testing, methanol, acetonitrile, acetone and other organic solvent precipitants required completely precipitated protein with a large volume and were unfavorable for sample testing of a low concentration. If treated with perchloric acid, the plasma would have more heteroatom peaks and greater interference. Using trichloroacetic acid not only used less volume, protein precipitation was also more complete without the interference of impurities. Thus, trichloroacetic acid was chosen as the protein precipitant.

## 3. Experimental Section

### 3.1. Materials

PLGA (Weight: ~60,000; lactide/glycolide ratio, 50/50) was purchased from Daigang Biological Co., Ltd. (Shandong, China). Lidocaine was obtained from Jinan Ruixing Medical Technology Co., Ltd. (Shandong, China). Bupivacaine was obtained from National Institute for the Control of Pharmaceutical and Biological Products (Beijing, China). All other materials and solvents were of reagent or analytical grade.

### 3.2. Microspheres Preparation

The oil-in-water (o/w) emulsion solvent evaporation method was applied to fabricate lidocaine–PLGA microspheres. Approximately 125 mg PLGA and 25 mg lidocaine were added to 2 mL of a mixture of dichloromethane:ethanol (3:1, *v*/*v*). After being completely dissolved, it was poured into 2% Tween-80 aqueous solution, and then, the mixture was emulsified by using a propeller stirrer at 500 rpm for 30 min. Then stirring at 300 rpm was continued for 6.5 h to evaporate the organic solvent. The hardened microspheres were filtered, rinsed with distilled water and dried under vacuum. 

### 3.3. Morphological Characterization and Particle Sizing

The morphological examination of the microspheres was performed using a Philips CM120 transmission electron microscope (TEM) (Philips, Amsterdam, The Netherlands). In practice, a drop of microspheres solution containing 0.1% (*w*/*v*) phosphotungstic acids was placed on a carbon film coated on a copper grid and observed at 80 kV in the electron microscope. 

The particle size distribution and mean diameter of the prepared lidocaine-loaded microspheres were determined by dynamic light scattering (DLS) using a NICOMP 380 Submicron Particle Sizer (Particle Sizing Systerms, Santa Barbara, CA, USA) equipped with a 5 mW HeNe laser at 632.8 nm. Sample solutions were transferred into the light scattering cells. The intensity autocorrelation was measured at a scattering angle of 90° at room temperature. Data were analyzed in terms of intensity-weighted NICOMP 380 Submicron Particle Sizer distributions. Each reported experimental result was the average of at least three *d*_h_ values obtained from the analysis of the autocorrelation function accumulated for at least 20 min. The zeta potential was measured on the same samples prepared for size analysis.

### 3.4. Drug-Loading Coefficient and Encapsulation Ratio

Drug-loading coefficient (DL%) and encapsulation efficiency (EE%) were calculated as described earlier. Firstly, lidocaine was extracted from the microspheres (10 mg) with dichloromethane (5 mL), and then, the extract solution was properly diluted prior to HPLC analysis. The content of lidocaine in the microspheres was determined by the HPLC method described below. Then, DL% and EE% were calculated according to Equations (1) and (2):

(1) $$DL\%＝\frac{W_{LID}}{W_{LID-MS}}\times 100\%$$

(2) $$EE\%＝\frac{W_{LID}}{W_{Total}}\times 100\%$$

(Note: *W*_LID_ represents the amount of lidocaine loaded in the microspheres, *W*_Total_ represents the total lidocaine amount added during preparation of the microspheres and *W*_LID-MS_ represents the weight of the lidocaine microspheres).

### 3.5. In Vitro Release

The *in vitro* release of lidocaine from microspheres was determined by the dialysis bag method [14,15,16]. The lidocaine microspheres (10 mg) were dispersed in 5 mL of PBS (pH 6.8) and placed into cellulose ester dialysis bags (Molecular Weight = 10,000). The dialysis bags were immersed in 45 mL release medium (pH 7.3 HSA) at 37 ± 0.5 °C with horizontal shaking at 50 rpm. Lidocaine solution (containing 167 μg) was also subjected to the release study to ensure that the diffusion of the lidocaine molecules across the membrane was not limited by the dialysis bag. At predetermined time points of 0.25, 0.5, 1, 2, 4, 6, 10, 14, 20, 30 and 40 h, 2 mL dissolution media were withdrawn and precipitated before HPLC analysis. The supernatant (10 μL) was then directly injected into the HPLC system and analyzed for the released lidocaine. The release profiles were plotted and fit using different *in vitro* release models.

### 3.6. Pharmacokinetic Study

Twelve rats were used in this experiment and randomly divided into two groups. On the testing day, 0.4 mL orbital blood samples were collected immediately before and at 0.083, 0.25, 0.75, 1, 2, 4, 8, 12 and 24 h after intravenous administration of lidocaine injection and microspheres. The plasma samples obtained were immediately centrifuged at 4000 rpm for 10 min. 200 μL of the supernatant were transferred to new glass tubes and stored at −20 °C. The plasma samples were directly precipitated before HPLC analysis. Briefly, 200 μL plasma were mixed with 200 μL trichloroacetic acid (TCA, 10%) and mixed for 2 min vigorously. The supernatant was collected after centrifugation at 12,000 rpm for 10 min, and 20 μL were injected into the HPLC system.

The pharmacokinetic parameters of each formulation were calculated by the non-compartmental method. The area under the curve and the mean residence time were determined by standard methods applying the linear trapezoidal rule. The maximum plasma concentration and time taken to reach the maximum plasma concentration were determined by a visual inspection of the experimental data. The elimination half-life (*T*_1/2_) was determined by linear regression of the terminal portion of the plasma concentration time data.

### 3.7. HPLC Analysis

HPLC analysis was performed using a Dikma Diamonsil C_18_ (5 μm, 200 × 4.6 mm) on a Shimadzu LC-20A HPLC system (Shimadzu Co., Tokyo, Japan) with an ultraviolet detector at room temperature. The wavelength of the ultraviolet detector was set at 263 nm. Methanol and 0.01 mol/L NaH_2_PO_4_ solution (30:70, *v*/*v*, pH = 2.0) were used as the mobile phase at a flow rate of 1 mL/min.

### 3.8. Pharmacodynamic Study 

Fifty rats were used in this experiment and randomly divided into five groups. The grouping and dosing of the rats are summarized in Table 3. Before the experiment, the rats were fasted overnight with free access to water. On the testing day, all of the rats were given different formulations by tail intravenous administration, as shown in Table 3. At 0.5, 1, 2, 4, 6, 8 and 10 h after intravenous administration, the rats were given 0.6% acetic acid by intraperitoneal injection. The aim of acetic acid was to establish the rat model of pain. Then, the number of twists of each rat was calculated in 15 min, and the response inhibition (RI) was evaluated according to the Equation (3) described below.

(3) $$RI\%＝\frac{N_{0}-N_{1}}{N_{0}}\times 100\%$$

(Note: *RI*, response inhibition; *N*_0_, the average number of twists in the control groups at each time point; *N*_1_, the average number of twists in the test groups at each time point) [17]. All experimental procedures were carried out in accordance with the guidelines of the Animal Care Committee of Hospital Laboratory Animal Center (2013 version).

**Table 3:** The grouping and dosing of lidocaine injection and microspheres.

Group	Formulation	Dose	Route of Administration
1 (control)	Normal saline	–	intravenous
2 (test)	Lidocaine injection	4.0 mg/kg	intravenous
3 (test)	Lidocaine microspheres	2.5 mg/kg	intravenous
4 (test)	Lidocaine microspheres	4.0 mg/kg	intravenous
5 (test)	Lidocaine microspheres	10.0 mg/kg	intravenous

### 3.9. Statistical Analysis

All data were presented as the mean ± standard deviations. Statistical significance was determined by Student’s *t*-tests with a *p*-value <0.05.

## 4. Conclusions

In this study, lidocaine microspheres were prepared by the o/w emulsion technique using PLGA for the controlled delivery. The average diameter of lidocaine PLGA microspheres was 2.34 ± 0.3 μm. The poly disperse index was 0.21 ± 0.03, and the zeta potential was +0.34 ± 0.02 mV. The encapsulation efficiency and drug loading of prepared microspheres were 90.5% ± 4.3% and 11.2% ± 1.4%. *In vitro* release indicated that the lidocaine microspheres had a well-sustained release efficacy, and *in vivo* studies showed that the area under the curve of lidocaine in microspheres was 2.02–2.06-fold that of lidocaine injection (*p* < 0.05). The pharmacodynamics results showed that lidocaine microspheres showed a significant release effect in rats. The process to achieve efficacy was calm and lasting. The analgesic effect had a significant dose-dependency.
